# *Citrullus colocynthis* (L.) Schrad (Bitter Apple Fruit): Promising Traditional Uses, Pharmacological Effects, Aspects, and Potential Applications

**DOI:** 10.3389/fphar.2021.791049

**Published:** 2022-01-25

**Authors:** Qin-Yuan Li, Mahzaib Munawar, Muhammad Saeed, Ji-Qiang Shen, Muhammad Sajjad Khan, Sobia Noreen, Mahmoud Alagawany, Muhammad Naveed, Asadullah Madni, Chang-Xing Li

**Affiliations:** ^1^ Department of Human Anatomy, Medical Institute of Qinghai University, Xining, China; ^2^ Department of Poultry and Animal Breeding, Faculty of Animal Production and Technology, The Cholistan University of Veterinary and Animal Sciences, Bahawalpur, Pakistan; ^3^ Faculty of Pharmacy, The Islamia University of Bahawalpur, Bahawalpur, Pakistan; ^4^ Department of Poultry, Faculty of Agriculture, Zagazig University, Zagazig, Egypt; ^5^ School of Pharmacy, Nanjing Medical University, Nanjing, China

**Keywords:** *Citrullus colocynthis*, traditional uses, health aspects, human, poultry

## Abstract

Health consciousness and increased knowledge about the side effects of synthetic drugs have enhanced interest in traditional medicines. Medicinal plants offer cures for various diseases, leading to improved living standards. This has brought ethnomedicinal studies into the spotlight and increased demand for herb-based medicines. *Citrullus colocynthis* is an herbaceous plant containing an abundance of nutrients that play a key role in the improvement of wellbeing. *C. colocynthis* has many biological properties, such as antioxidative, hypoglycemic, antibacterial, anti-cancerous, anti-inflammatory, analgesic, gastrointestinal tract, reproduction, protection, anti-microbial, antidiabetic, hypolipidemic, antineoplastic, profibrinolytic, anti-allergic, pesticidal, and immune-stimulatory. There are numerous bioactive compounds like cucurbitacin, flavonoids, and polyphenols in *C. colocynthis* that give it medicinal properties. Herein, we have extensively compiled, reviewed, and analyzed significant information on *C. colocynthhis* from the best published available evidence in PubMed, Scopus (Embase), Web of Science (Web of Knowledge), Cochrane Library, and Google Scholar, etc. Scientific literature evidenced that owing to the bioactive constituents, including cucurbitacin, polyphenols, flavonoids, and other potent molecules, *C. colocynthis* has many pharmacological and physiological functions. It possesses multi-beneficial applications in treating various disorders of humans and animals. So, the primary purpose of this comprehensive review is to provide an overview of the findings of positive impacts and risks of *C. colocynthis* consumption on human health, especially in poultry and veterinary fields. In the future, this narrative article will be aware of discoveries about the potential of this promising natural fruit and its bioactive compounds as the best nutraceuticals and therapeutic drugs in veterinary and human medicine.

## 1 Introduction

Plants have supplied many essential human needs, including a variety of therapeutic medications (Alagawany et al., 2020, 2021a, b; Dhama et al., 2021). Therefore, deliberate efforts towards cultivation are crucial for the continuous availability of those plant species. Medicinal plants have been used in healthcare for a long time, and their use to prevent and treat illness is expanding worldwide (Dhama et al., 2018; Bilal et al., 2021; Reda et al., 2021; Saeed et al., 2021). The medicinal properties of plants are due to the natural chemicals/compounds they contain (Saeed et al., 2019; Alagawany et al., 2021c; Garg et al., 2021; Zhang et al., 2021). Plants are a source of food and act as raw materials from which a variety of drugs are synthesized (Hassan, 2012). *Citrullus colocynthis* is a desert plant and a source of several bioactive compounds such as essential oils, glycosides, flavonoids, alkaloids, and fatty acids*.* Medicinal plants improve the immune system. The dried fruit pulp of *C. colocynthis* has been used to treat gastrointestinal disorders like indigestion, gastroenteritis, and intestinal parasites. *C. colocynthis* also has excellent pharmacological properties, such as being a laxative and purgative; it is anti-diabetic, anti-inflammatory, anthelmintic, and anti-cancerous. The fruit has been studied extensively for its antimicrobial, antioxidant, and anti-inflammatory activities (Hussain et al., 2014). *C. colocynthis* seed powder (CCSP) has been used as an emulsifier, fat binder, and flavoring (De Smet., 1997). *C. colocynthis* has also long been utilized in popular cuisine. Some of its medicinal characteristics include antioxidant, anti-inflammatory, anti-diabetic, and antibacterial activities (Kamran et al., 2018). Its pharmacological properties include antioxidative, hypoglycemic, antibacterial, anti-cancerous, anti-inflammatory, analgesic (Sanafi et al., 2006). *C. colocynthis* has antidiabetic, hypolipidemic, antineoplastic, profibrinolytic, antiallergic, antimicrobial, pesticidal, and immune-stimulatory effects. It also affects the reproductive system and fertility (Meybodi, 2020). *C. colocynthis* acts as an antioxidant and anesthetic in humans (Hyderi et al., 2015); its oil can be used to treat constipation (Qureshi et al., 2010), while an extract showed anti-tumor activity on cancerous cells (Abdulridha et al., 2020) and its leaves are anti-cancerous and anti-adipogenic (Perveen et al., 2020). Phytochemical screening of *C. colocynthis* fruit extract revealed anti-diarrheal properties (Dhakad, 2017). The irregular use of antimicrobials results in drug resistance in animals and humans, adversely affecting their health. Therefore, in 2006, the European Union prohibited antibiotics as growth promoters (Milanov et al., 2016). Due to this restriction, many alternative antimicrobials are being used, and preferences trend towards photogenic products extracted from herbs and spices with known antimicrobial properties (Bajagai et al., 2020). Many other products have been selected as alternatives to antibiotic growth promoters; these include probiotics, prebiotics, enzymes, organic acids, acidifiers, antioxidants, and phytogenic additives (Perić et al., 2009).

In Pakistan, the poultry industry is a key sub-sector of the livestock industry, with current investment of >750 billion and a growth rate of 7.5% per annum. Pakistan is the 11th largest poultry producer globally, with an estimated population of 64.01 million layers, 1,407.73 million broilers, and 14.34 million breeders (Pakistan economic survey, 2020). This indicates the strong growth and importance of, as well as prospects for broiler farming in Pakistan. The antimicrobial growth promoters boost feed conversion and body weight gain as they change the composition and activity of gut microflora (Al Dobaib and Mousa, 2009). The focus of broiler production is growth and performance, and the latter and health depend on the microflora present in the lower gastrointestinal tract (GIT) of broiler chicken (Rinttilä and Apajalahti, 2013). Change or imbalance in gut microbiota can adversely affect nutrient utilization and gut health (Choct., 2009). Phytobiotics are natural, less toxic, and residue-free. Growth promoters improve digestive capacity and growth, increase nutrient availability, and reduce potential pathogens in the GIT (Yitbarek, 2015). These additives also improve feed intake, thus improving the feed conversion and weight gain of broiler chickens (Ertas et al., 2005). Phytobiotics are added to poultry feed and are considered an antimicrobial substitute. These compounds can be used as replacements for antibiotic growth promoters because of their antibacterial, antifungal, antiparasitic, and immune stimulatory attributes, resulting in improved product performance of chickens (Abd El Ghany and Yazar Soyadı, 2020). Ten bioactive components were isolated from *C. colocynthis* seeds (CCS). CCS are anti-microbial, immune-stimulating, and enhance growth. CCSP improves production performance and alleviates immune suppression (Alzarah et al., 2021). CCS contains 13.5% protein, is rich in methionine and cysteine, and is limited in lysine. The *in vitro* digestibility of seed protein is 75.9% (Sawaya et al., 1986).

Previous research has reported multiple benefits of *C. colocynthis* for humans, livestock, and poultry. This literature, from various sources, has been reviewed. As the literature on the use of *C. colocynthis* in poultry and its importance in humans is limited, we recommend further research on it and its extract in manufacturing poultry and human medicine. We aim to broaden the scope of *C. colocynthis* use and increase the awareness of scientists and veterinarians regarding the benefits of this plant for human and poultry health.

## 2 Botanical Description

*C. colocynthis* is a perennial plant with perennial roots and angular, tough, rough, and vine-like stems that spread on the ground and may climb up from there. They produce a single yellow flower at leaf axils. They are monoecious and have long peduncles and tuberous rootstock sprouting long trailing or climbing stems (Pravin et al., 2013) (Table 1).

**TABLE 1:** Botanical description of *C. colocynthis* (Pravin et al., 2013).

Botanical description	
Roots and stem	Perennial roots, stems are angular, tough, and rough vine-like that spread on the ground and may climb up
Seeds	Yellow to brown in color, smooth in texture, and oval in shape
Flowers	A single yellow color flower at leaf axils. They are monoecious and have long peduncles
Leaves	Angular and about 5–10 cm long. They are triangular, rough, and green
Fruit	15-30 fruits that are about 7–10 cm in diameter. Color may be yellow or green with yellow stripes. Fruit pulp contains oval seeds

**FIGURE 1:**
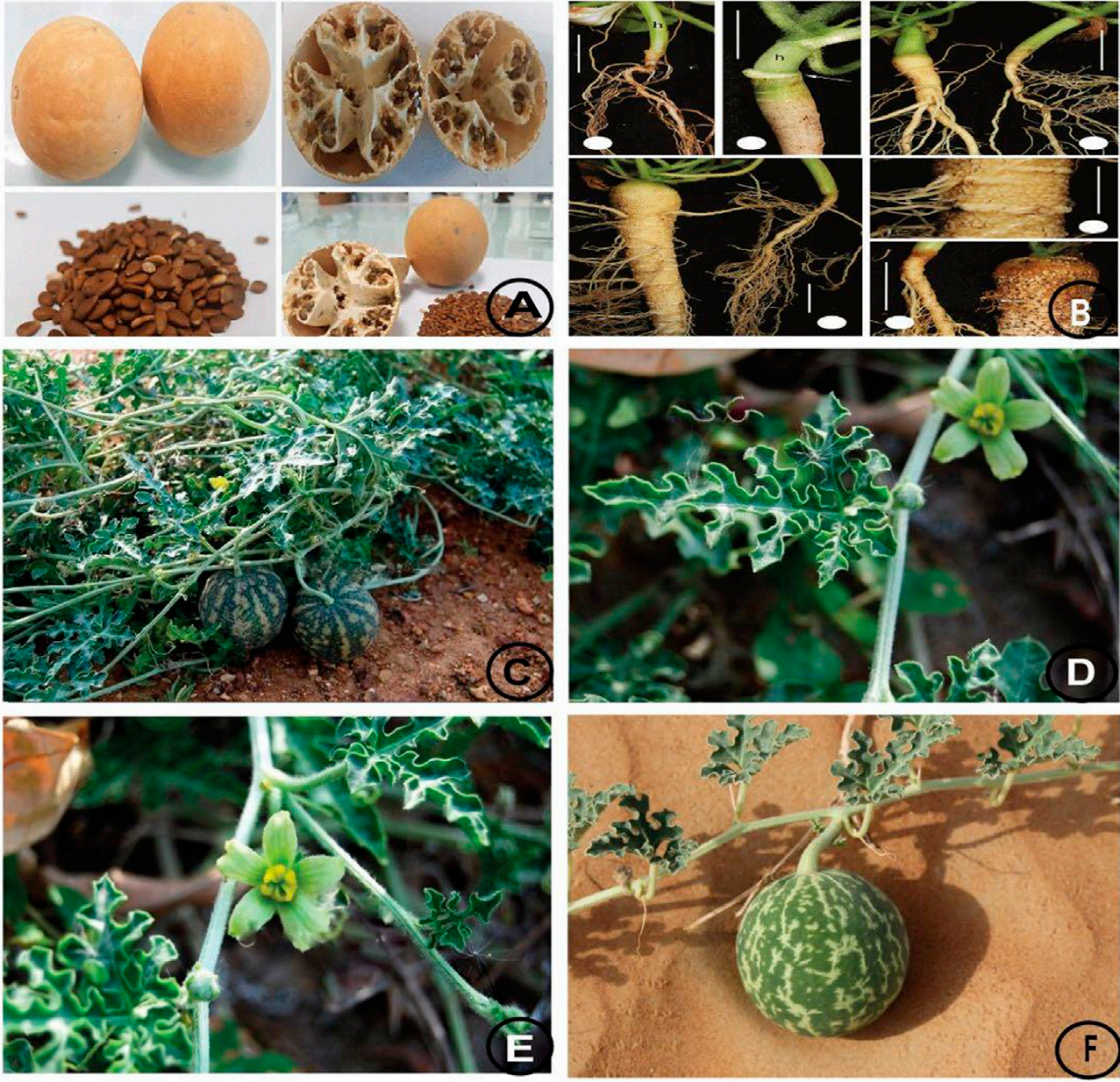
**(A)** Seeds **(B)** roots **(C)** plant **(D)** leaf **(E)** flower and **(F)** fruit of C. colocynthis.

*C. colocynthis* is a desert plant, as shown in Figure 1

## 3 Geographic Distribution

The plant is native to the arid sandy areas of West Asia, Arabia, tropical Africa, and the Mediterranean (Pravin et al., 2013). It is also widely distributed in the desert area of Pakistan (Kamran et al., 2018). *C. colocynthis* originated in Asia and the Mediterranean Basin, particularly Turkey and Nubia, to the western coastal regions of Africa, the Sahara, and Egypt in the east. It is also found in India and the northern coastal regions of the Caspian and Mediterranean seas. *C. colocynthis* belongs to the Cucurbitaceae family, and its common names are shown in Table 2.

**TABLE 2:** Common names used for *C. colocynthis* (De Smet., 1997; Elltayeib et al., 2020; Pravin et al., 2013).

Common names	Languages
Colocynth	English
Bitter gourd	
Bitter apple	
Bitter cucumber	
Koloquinthe	German
Coloquinte	French
Indravaruni	Sanskrit
Handhal	Arabic
Ghurunba/Kortuma	Punjabi
Makhal	Bengali
Paedikari Attutummatti	Tamil
Kadu indravani	Marathi
Indrayan	Gujarati
Paikummatti	Malyalam
Indrayan	Hindi
Maraghonae	Pashto

### 3.1 Proximate Composition

The proximate composition of *C. colocynthis* is given in Table 3. The proximate analysis of *C. colocynthis* revealed 24.37% protein, 1.91% fiber, 10.88% carbohydrate, 56.61% fat, 3.15% ash, and 3.08% moisture (Ogundele et al., 2012).

**TABLE 3:** Proximate composition of *C. colocynthis* (Akpambang et al., 2008).

Parameter	*Citrullus colocynthis*
Protein	25.73b ± 0.06
Fat	46.24c ±0.02
Moisture	4.85a±0.04
Ash	4.48b ± 0.02
Fiber	5.00b ± 0.07
Carbohydrate	13.70b ± 0.02

## 4 Traditional Uses

**FIGURE 2:**
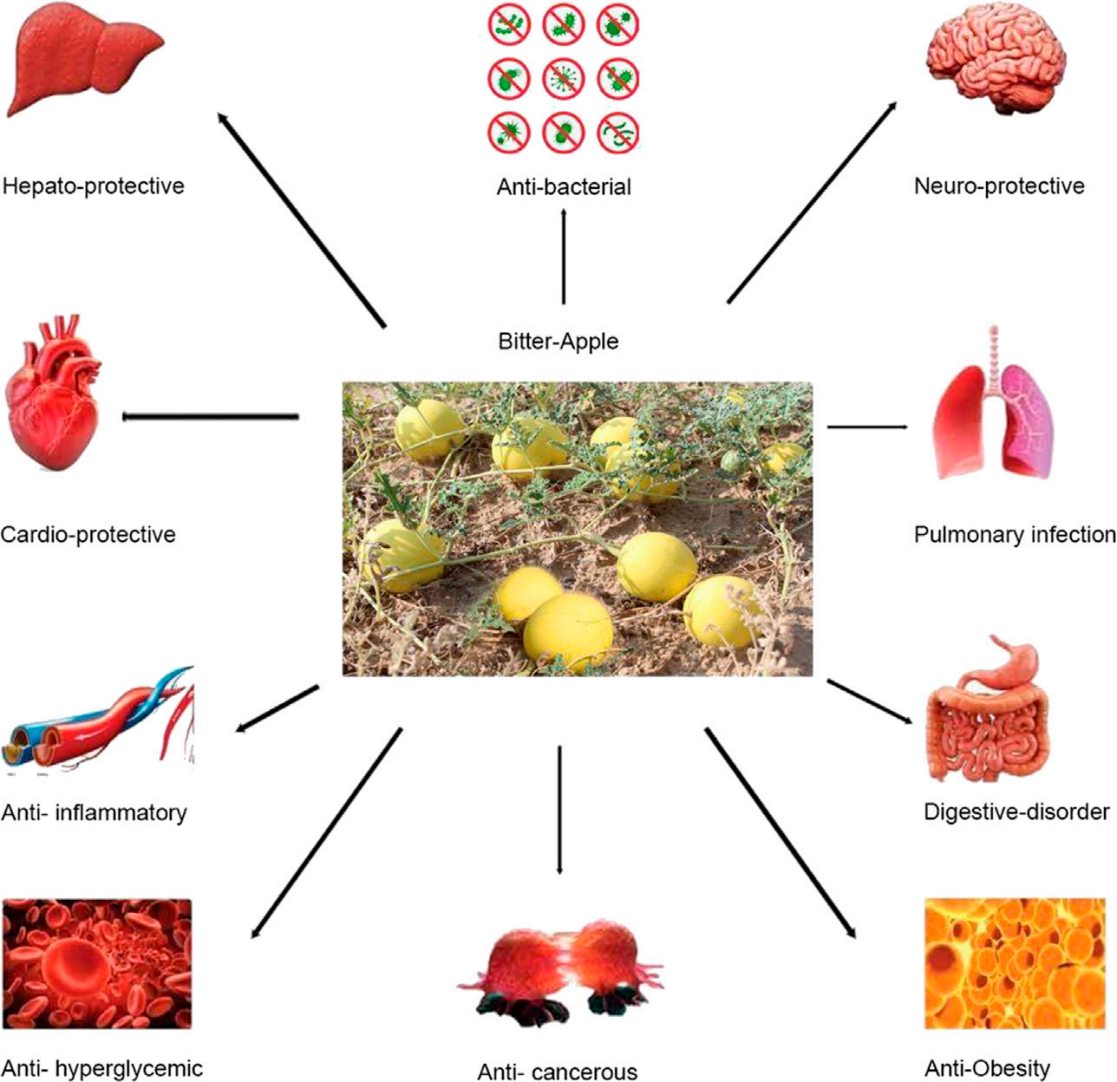
Promising pharmacological effects of *C. colocynthis.*

*C. colocynthis* can be used to treat gastrointestinal conditions and pulmonary, skin, and bacterial infections (Hameed et al., 2020); constipation; edema, cancer, and diabetes (Kumar et al., 2008). The dried pulp of the fruit of *C. colocynthis* is used as a remedy for gastrointestinal disorders like indigestion, gastroenteritis, and intestinal parasites (Hussain et al., 2014). The plant is also used to treat diabetes, liver problems, weak bowel movements, and obstruction or paralysis of the intestine (Rahimi et al., 2012). The fruit extract is used as an analgesic (Heydari et al., 2015). The vital pharmacological effects of *C. colocynthis* are shown in Figure 2.

## 5 Phytochemistry

*C. colocynthis* contains several bioactive compounds like cucurbitacin, flavonoids, and polyphenols, which impart medicinal properties (Bhasin et al., 2020). The phytochemical constituents of *C. colocynthis* are shown in Table 4.

**TABLE 4:** Chemical constituents of *C. colocynthis* (Al-Snafi, 2016).

❖Carbohydrates
❖Alkaloids
❖Proteins
❖Glucosides
❖Phenolics, flavonoids
❖Tannins
❖Saponins
❖Cardiac glycolipids
❖Flavone, terpenoids
❖Cucurbitacins
❖Anthranol
❖Saponarin
❖Steroids
❖Trace elements

Three flavone glucosides–isovitexin, isosaponarin, isoorientin, and the two cucurbitacin glucosides 2-glucopyranosyl-cucurbitacin L and glucopyranosyl cucurbitacin were extracted from the fruits of the locally growing *C. colocynthis* and identified. The flavonoids were shown to have considerable antioxidant effects, which is a key characteristic for treating various disorders because reactive oxygen species play an important role in inflammation, cancer, tissue damage, and a variety of diseases (Delazar et al., 2006). Phytochemical screening also revealed the presence of tannins, flavonoids, alkaloids, saponins, and glycosides in *C. colocynthis*. The chemical components of the ethanolic extract of *C. colocynthis*, including alkaloids, glycosides, and flavonoids, could have a strong antibacterial effect (Najafi et al., 2010). Terpenoids, steroids, alkaloids, flavonoids, glycosides, phenols, tannins, flavones, and saponins were found in crude extracts of *C. colocynthis* (Ahmed et al., 2019). Carbohydrates, proteins, tannins, distinct amino acids, steroids, phenolic compounds, alkaloids, glycosides, terpenoids, and cucurbitacins A, B, C, D, E, J, and L were also all found in various preparations of *C. colocynthis* (Mazher et al., 2020).

### 5.1 Bioactive Compounds and their Structure-Activity Relationship

#### 5.1.1. Cucurbitacin

**FIGURE 3:**
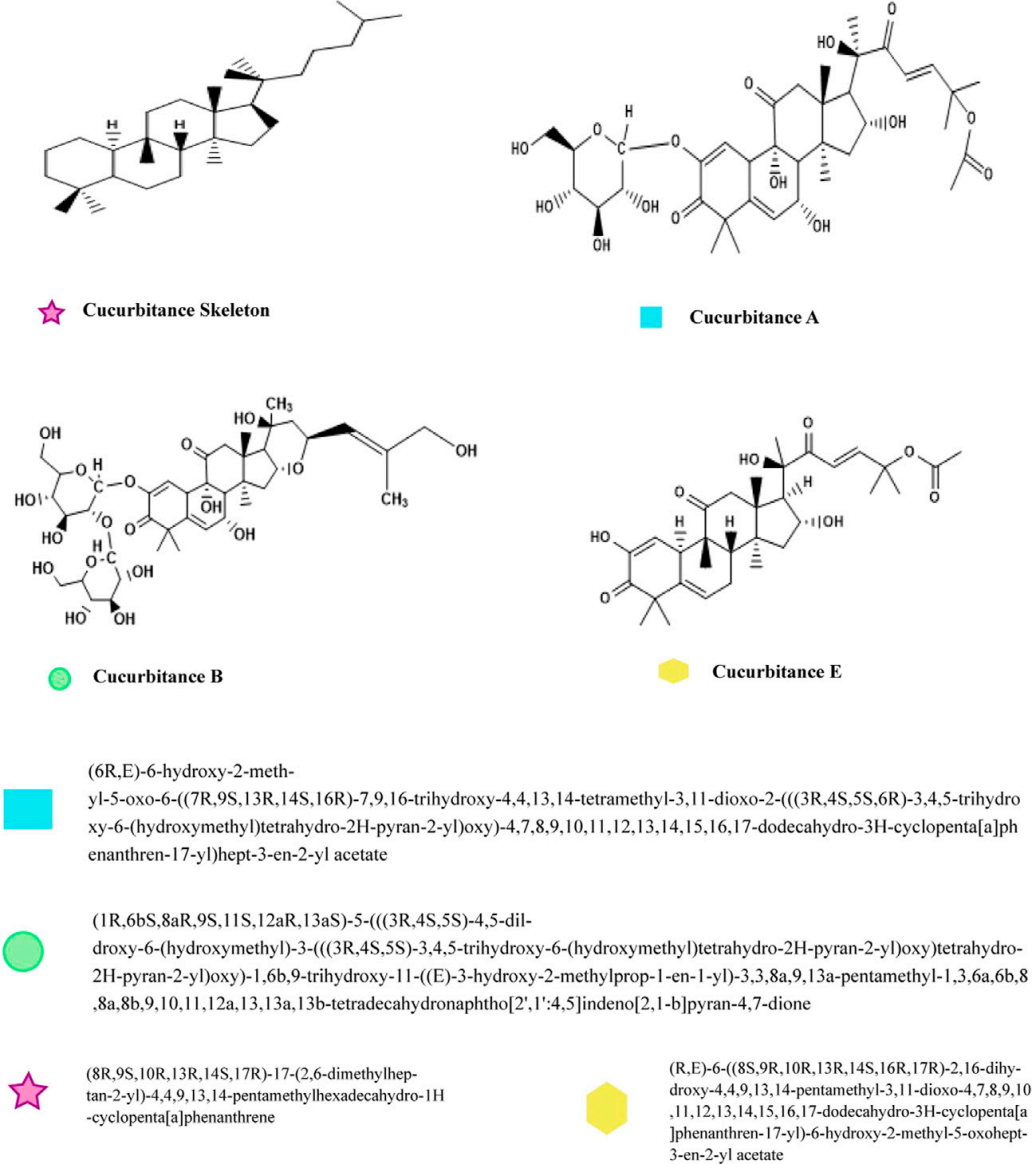
Chemical structure of different Cucurbitacin.

Colocynthosides A, cucurbitacin L, and cucurbitacin B were isolated from the fruit of *C. colocynthis*. The main cucurbitane-type triterpene glycoside and its aglycon, Cucurbitacin E 2-O—D-glucopyranoside, and cucurbitacin E, showed anti-allergic properties (Yoshikawa et al., 2007). Natural cucurbitacins are triterpenoid chemicals famous for their bitter taste and toxicity. Due to their cytotoxic activities, cucurbitacins play an important role in drug discovery, particularly in anticancer drug development (Chen et al., 2005). Figure 3 shows the structure of various Cucurbitacin.

The structure-activity relationship of the anti-cancerous effects of cucurbitacin and their derivatives, which are capable of electrophilic attack on cellular structures or genetic material, have been studied. This could be used to derive new anti-cancerous agents (Lang et al., 2014).

### 5.2 Glycosides, Flavonoids, and Phenolic Acids

*C. colocynthis* fruit contained 2-O—D-glucopyranosyl-Cucurbitacin L, 2-O—D-glucopyranosyl-Cucurbitacin I and isosaponarin. Kaempferol, quercetin, myricetin, catechin, gallic acid, vanillic acid, p-hydroxybenzoic acid, p-coumaric acid, caffeic acid, sinapic acid, chlorogenic acid, and ferulic acid were also found in *C. colocynthis* (Delazar et al., 2006; Hussain et al., 2014). Flavonoid C-glycosides show considerable anticancer and antitumor action and antibacterial, antifungal, antioxidative, anti-diabetic, anti-inflammatory, antiviral, and hepatoprotective activities, among other biological benefits (Xiao et al., 2016). The structure-activity relationship for quercetin and structurally similar flavonoids has a strong tumor necrosis factor-alpha inhibitory effect and a positive chemical potential and negative electrophilicity index that was considered beneficial (Geoffrey et al., 2020).

### 5.3 Fatty Acids

**FIGURE 4:**
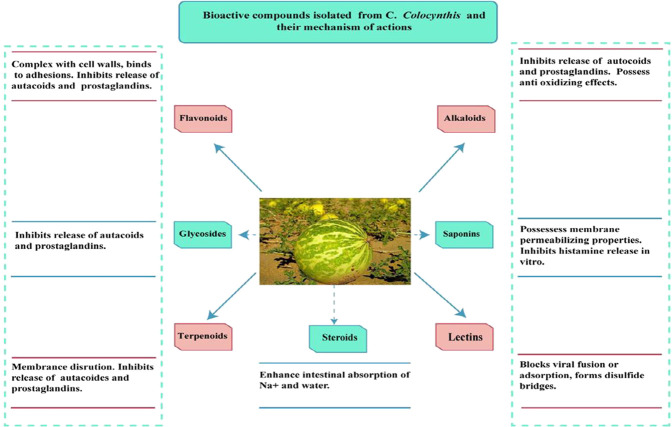
Phytoconstituents of *C. Colocynthis* and their mechanism of action.

Stearic, linolenic, oleic, linoleic, myristic, and palmitic acids were present in CCS (Gurudeeban et al., 2010). Figure 4 shows various phytochemicals along with their mechanism of action.

## 6 Medicinal Properties

The bitter and spicy *C. colocynthis* fruit is used to treat colds, diarrhea, parasitic worms, the expulsion of wind, tumors, ascites, leukoplakia, ulcers, asthma, bronchitis, diabetes insipidus, jaundice, splenomegaly, neck tuberculosis, constipation, anemia, throat diseases, elephantiasis, and joint pain; it is also used as an antipyretic. The root can be used to treat jaundice, ascites, urinary disorders, rheumatism; in children, and it can be used against enlarged abdomens, coughs, and asthma attacks. A root plaster can also be used to treat breast inflammation. An application of the fruit or root with a mixture of water and/or Nux vomica can treat papules and acne (Pravin et al., 2013). Different studies on *C. colocynthis* have been summarized in Table 5.

**TABLE 5:** Biological effects and health benefits of *C. colocynthis*.

Plant part	Traditional use	Specie	References
Dried fruit pulp	Gastrointestinal disorders	Human	Hussain et al*.* (2014)
Fruit	Antioxidant, antimicrobial, and anti-inflammatory	Human	Hussain et al*.* (2014)
Fruit	Cold, diarrhea, deworming, antipyretic, expulsion wind, treatment of tumors, ascites, leukoplakia, ulcers, asthma, bronchitis, diabetes insipidus, jaundice, splenomegaly, neck tuberculosis, constipation, anemia, diseases of the throat, elephantiasis, and joint pain	Human	Pravin et al. (2013)
Root	Jaundice, ascites, diseases of the urinary system, rheumatism, and to treat an enlarged abdomen, and cough	Human	Pravin et al. (2013)
Ethanolic extract of the *C. colocynthis*/aqueous and diluted acetone extracts/ethyl acetate extract from leaves	Anti-bacterial	Bacteria (Gram-positive and Gram-negative)	Hameed et al. (2020), Khatibi and Teymorri. (2011), Chawech et al. (2015)
Ethanol extraction of *C. colocynthis*	Anti-hyperglycemic	Rat	Oryan et al. (2014)
Seed	Anti- Heat stress	Poultry	Alzarah et al. (2021)
Hydroalcoholic peel extract	Cardioprotective	Rabbit	Tabani et al. (2018)
Hydro-alcoholic leaf extract	Anti-hyperglycemic and anti-hyperlipidemic	Rat	Ebrahimi et al. (2016)
Colocynth oil	Anti-obesity	Rat	Meziane et al. (2012)
The ethanol extract of *C. colocynthis* fruit	Anti-mycotic	Plant pathogenic fungi	Hadizadeh et al. (2009), Hameed et al. (2020)
Ethanol extract of *C. colocynthis*	Antifertility	Rat	Chaturvedi et al. (2003)
*C. colocynthis* plant	Diabetes, liver problems. weak bowel movements, Obstruction, or paralysis of the intestine		Rahimi et al. (2012)
Fruit extract	Analgesic	Human	Hyderi et al. (2015)
Leaves	Anticancerous, Anti-adipogenic, and hypolipidemic	Human	Perveen et al. (2020)
*C. colocynthis*	Anti-fertility	Human	Amal et al. (2016)
*C. colocynthis* fruit extract	Anti-tumor	Human cell lines	Saeed et al. (2019)
*C. colocynthis* fruit	Anthelmintic	Animals	Qureshi et al. (2010)
*C. colocynthis* oil	Constipation	Human	Qureshi et al. (2010)
*C. colocynthis* extract	Anti-cancer	Human	Abdulridha et al. (2020)
*C. colocynthis* pulp and seed	Anti-diabetic	Rabbit	Shafaei et al. (2014)
*C. colocynthis* fruit	Anti-diabetic	Human	Huseini et al. (2009)
Hydro-alcoholic *C. colocynthis* fruit extract	Anti-diarrheal	Rat	Dhakad. (2017)
Hydro-ethanolic pulpy flesh of *C. colocynthis* with its seeds	Anti-hyperglycemic	Rat	Ghauri et al. (2020)
*C. colocynthis* extract	Anti-oxidant		Benariba et al. (2013)
*C. colocynthis* fruit extract	Anti-convulsant	Rat	Mehrzadi et al. (2016)
Leaf and root extract	Skin disorders	Humans	Upadhyay et al. (2007)
Roots paste	Joint problems	Humans	Upadhyay et al. (2007)
*C. colocynthis*	Anti-obesity	Rat	Sari et al. (2019)

## 7 Pharmacological Effects of *C. Colocynthis*

*C. colocynthis* has many therapeutic uses and has also been studied for its various pharmacological effects. It is considered an excellent therapeutic agent for the trachea, gut, and cardiovascular system (Hussain et al., 2014).

### 7.1 Antimicrobial Properties

Previous studies report that aqueous and diluted acetone extracts (from the plant’s roots, stems, leaves), and three maturation stages of its fruit and seeds of *C. colocynthis* plant are active against Gram-positive and Gram-negative bacteria (*Escherichia coli, Pseudomonas aeruginosa, Staphylococcus aureus,* and *Enterococcus faecalis*), but have a more substantial effect on newer bacteria. The broth dilution method measured the minimal inhibitory concentration (MIC) preventing visible bacterial growth. MIC was tested for concentrations ranging from 0.10 to 6.50 mg/ml. For aqueous extracts of immature fruits, the MIC was 0.20 mg/ml for *Escherichia coli, Pseudomonas aeruginosa.* The activity depends on the strains, plant organs, stage of maturity, and the nature of the extraction (Marzouk et al., 2009).

The effect of the ethanolic extract of the *C. colocynthis* fruit was studied by the well diffusion method and disc diffusion method, and results showed that it has a standard antibacterial effect on both Gram-positive bacteria (*S*. *aureus* and *Bacillus subtilis*) and Gram-negative bacteria like *Klebsiella pneumoniae*. However, the ethanolic extract from the pulp was more active against Gram-positive bacteria, while the seed extract was slightly less effective against both types of bacteria (Hameed et al., 2020). Both aqueous and diluted acetone extracts showed antibacterial effects against both Gram-positive and Gram-negative bacteria as evaluated by *in vitro* study; the best results came from an aqueous fruit extract and poorest from a root extract. Immature seeds and fruits showed the highest antibacterial activity. The highest MICs were obtained from the fruit aqueous extracts 0.20 mg/ml against *E. coli* and *P. aeruginosa* (Khatibi and Teymorri, 2011). The ethyl acetate extract of the leaves showed promising results against Gram-positive and Gram-negative bacteria (*Salmonella enteritidis, B. cereus, Escherichia coli, S. aureus, Enterococcus faecalis,* and *P. aeruginosa*) using the agar disc well-diffusion method. MIC values were obtained for the ethyl acetate extract 0.625 mg/ml against *Bacillus cereus* (Chawech et al.,. 2015).

### 7.2 Anti-Fungal Properties

The aqueous plant extract and a diluted acetone extract of *C. colocynthis* (roots, stems, leaves, and fruit and seeds at various stages of maturity) were selected for testing against several strains of *Candida* (*Candida glabrata, C. albicans, C. parapsilosis,* and *C. kreusei*). In a water extract, the mature fruit had the greatest anti-*Candida* effect among all strains (MIC 0.20 mg/ml); the immature fruit was the most active in an acetone extract against all strains (Marzouk et al., 2009). Using an ethanol extract of *C. colocynthis* fruit on various fungal species (*Fusarium oxysporum, C. albicans, Aspergillus fumigatus,* and *A. niger*) were assessed *in vitro* and produced good results against all strains, particularly *C*. *albicans*. The efficiency of the extracts was enhanced by increasing the concentration thereof. The results showed that all fungal strains were sensitive against the extracts of the fruit pulp, seeds, and roots of *C. colocynthis* (Hameed et al., 2020).

The anti-mycotic activity of the ethanol extract of *C. colocynthis* fruit was tested against pathogenic plant fungi using the agar dilution method and showed promising results. An organic extract of *C. colocynthis* fruit can thus be used as to alternative synthetic fungicide in agro-industries (Hadizadeh et al., 2009).

### 7.3 Antioxidant Effects

The methanolic fruit extract of *C. colocynthis* was found to be a good antioxidant. It exhibited good free radical scavenging activity due to the presence of gallic acid, a phenolic compound. The highest antioxidant and free radical scavenging ability of the fruit extract was observed at a concentration of 2,500 mg ml^–1^ (Kumar et al., 2008). Cucurbitacin is also an effective antioxidant. that can eliminate free radicals like hydroxyl radicals, superoxide anions, and singlet oxygen. It can also completely inhibit lipid peroxidation and oxidation (Bernard and Olayinka, 2010). Phytochemical screening of *C. colocynthis* extracts revealed that the natural compounds present therein make it an excellent antioxidant (Benariba et al., 2013). *C. colocynthis* oil can boost the function of antioxidant enzymes and protect the liver from injury (Amamou et al., 2015). An *in-vitro* study states that *C. colocynthis* can prevent the damage caused by free radicals to the body. Various biochemicals in *C. colocynthis* make it a good antioxidant (Rizvi et al., 2018).

### 7.4 Anti-Inflammatory and Analgesic Properties

*C. colocynthis* water extracts were found to possess anti-inflammatory and analgesic activities. All extracts displayed palliative and anti-inflammatory potential at unique doses despite causing acute toxicity. The outcomes of the problem were acquired from unripe fruits and seeds. Stem and root extracts reduced big inhibitory endeavors in analgesic and anti-inflammatory models (Marzouk et al., 2009). The main bioactive chemical components in the chloroform part of CCS extracts came from the separation and characterization of glycoside 11-deoxycucurbitacinI2ObD at the doses of 0.5 and 1 mg/kg body weight in two animal models. The compounds studied demonstrated strong analgesic and anti-inflammatory effects in two animal models (Marzouk et al., 2013). The *in vivo* analgesic and anti-inflammatory actions of organic extracts of unripe fruits and seeds of Tunisian melon were studied. All extracts showed marked analgesic and anti-inflammatory effects at different doses. *C. colocynthis* Schrad appeared to interfere with histamine and serotonin pathways and strongly interfered with prostaglandin and kinin-like pathways (Marzouk et al., 2011). The methanolic extract of *C. colocynthis* leaves was evaluated for anti-inflammatory activity using different *in vivo* screening models. It had an inhibitory effect on the edema of the paw caused by different inflammatory drugs at the doses of 250 and 500 mg/kg, the infiltration of leukocytes, and the formation of exudate caused by carrageenan, thus presenting an anti-inflammatory effect on the acute and subacute phases of inflammation (Rajamanikam et al., 2010).

### 7.5 Anti-Hyperglycemic Activity

Various extracts of *C. colocynthis* peel–aqueous, alkaloidal, saponin, and glycosidic–were examined for their effects on plasma glucose levels in rabbits. The activity of the saponin extract on fasting blood sugar levels of alloxan-induced diabetic rabbits was examined. Normal rabbits orally (300 mg/kg) administered with an aqueous extract of *C. colocynthis* showed noticeably low plasma glucose levels after 1 h; this increased to high levels after 2, 3, and 6 h. The saponin extract lowers the fasting glucose levels after 1 and 2 h and considerably after 3 and 6 h (Abdel Hassan et al., 2000). The ethanol extract of *C. colocynthis* at the dose rate of 300 mg/kg on the blood glucose attention within the alloxan brought about diabetes in rats. The results showed that *C. colocynthis* could lower blood glucose markedly in contrast to manipulating the diabetic team. CCS were also shown to have a marked anti-hyperglycemic effect, supporting the everyday use of *C. colocynthis* to treat diabetes mellitus (Oryan et al., 2014). Wistar rats and streptozotocin-diabetic rats were injected with various extracts of CCS (total alkaloids, aqueous, saponin, and glycosidic) intraperitoneally to examine their anti-hyperglycemic activity. The results showed that these extracts had a good anti-hyperglycemic effect on the diabetic rats and stabilized the blood glucose of the control rats to within a normal range. The aqueous extract 2.5 g/kg (BW) showed the highest activity by decreasing the blood glucose level (Lahfa et al., 2017). The hydro-ethanolic pulpy flesh of *C. colocynthis* also demonstrated an exceptional anti-hyperglycemic effect in a diabetic rat at the dose rate of 300 mg/kg by decreasing its blood glucose and triglyceride, and cholesterol levels. *In vitro* testing also showed that *C. colocynthis* inhibited glucosidase, which is responsible for postprandial hyperglycemia, strongly indicating that it is a potential candidate for a hyperglycemia treatment (Ghauri et al., 2020).

The *C. colocynthis* fruit possesses insulin-enhancing activity. This activity may explain in part its antidiabetic effects in traditional medicine. It also identifies the *C. colocynthis* as a source of a potential novel insulin enhancer that may prove to be useful to reduce hyperglycemia in type 2 diabetes. The ethyl acetate fractions of aqueous non-defatted seed and pulp extracts were used. Two extracts enhanced the insulin-induced translocation of glucose transporter (GLUT4) from intracellular storage sites towards the plasma membrane and accordingly increased insulin-induced glucose uptake. Several of our findings suggested that pulp extract, which increased glucose uptake more than its seed homolog, increased GLUT4 translocation and glucose uptake by acting on the same intracellular signaling cascade as the one employed by insulin (Drissi et al., 2021).

### 7.6 Anti-Obesity Activity

Results from the administration of 4%colocynth oil to the offspring of overweight rats suggest that it can aid in weight reduction, maintenance of a healthy lipid profile, and controlling glucose levels. This suggests that the oil has a remedial and regulating effect on obesity (Meziane et al., 2012). The effect of glycoside and alkaloid extracts of colocynth were studied on 26 adult male Wistar rats. Animals administered with alkaloids showed weight regression, while those given glycosides were appropriately sized, starting from the 6th week. It became a widespread give-up of treatment (Tabani et al., 2018). These results suggest that *C. colocynthis* seed oil has good potential for treating obesity and related problems (Sari et al., 2019).

### 7.7 Anti-Tumor Activity

The anti-tumor activity of *C. colocynthis* can be attributed to different pathways and properties, including apoptotic pathways, antioxidant and anti-inflammatory effects, inhibition of the Wnt/ß-catenin signaling pathway, and anti-metastatic effects. The cucurbic acid in *C. colocynthis* gives the plant its anti-cancerous properties (Abdulridha et al., 2020). The methanolic extract of *C. colocynthis* leaves and its two fractions, ethyl acetate and chloroform, possess notable anti-cancerous effects on the human breast cancer cell line. Bioassays showed a marked reduction in the multiplication and growth of cells treated with these extracts compared to untreated cells. The presence of cyclin-CDK inhibitors means that *C. colocynthis* extract can arrest human breast cancer cells (Perveen et al., 2021). Colocynth fruit pulp extracts can also block the proliferation and metastatic activity of breast cancer cells and prevent cell migration, the induction of cell apoptosis and cell proliferation, and inhibit cancer stemness properties in breast cancer cells (Chowdury et al., 2017). By modulating the metabolism of lipids, *C. colocynthis* leaves showed excellent potential as anti-cancerous agents in treating human breast cancer (Perveen et al., 2020). The extract of *C. colocynthis* fruit also showed anti-tumor activity on cancerous cell lines (Saeed et al., 2019).

### 7.8 Hepatoprotective Activity

The glycoside and alkaloid extract of colocynth (70 mg/kg single intraperitoneal injection) were analyzed for their effect on metabolic and histological liver disorders in Wistar rats. Treatments therewith showed hypoglycemic, lipid-lowering, and hepatoprotective effects. There was a marked increase in the levels of the liver function markers aspartate aminotransferase, ALT, and alkaline phosphatase (Tabani et al., 2018). The administration of ethanolic extracts of *C. colocynthis* (200 mg/kg BW), as opposed to paracetamol, resulted in hepatotoxicity in albino rats. The 90% ethanolic extract of *C. colocynthis* leaves exhibited *in-vivo* hepatoprotective effects that can be attributed to cell membrane stabilization and liver cell regeneration (Dar et al., 2012).

The hydro-alcoholic extract of *C. colocynthis* leaves (75 mg/kg body weight orally for 3 weeks) showed good anti-hyperglycemic and anti-hyperlipidemic effects. In addition, *C. colocynthis* leaf extract might also have a protective effect on the liver, as demonstrated by the markedly lower fasting blood sugar, low-density lipoprotein, cholesterol, alanine aminotransferase, creatinine, aspartate aminotransferase, urea, triglycerides, and bilirubin levels in diabetic rats to which it was administered (Ebrahimi et al., 2016).

### 7.9 Cardioprotective Activity

Experiments on male rabbits suggest that the administration of adrenaline prompted myocardial damage, as shown by the increased ranges of histomorphological adjustments in the myocardium associated with free radical manufacturing in cardiac tissue. *C. colocynthis* provided cardiac protection by decreasing oxidative stress caused by the experimental myocardial infarction, preventing the free radical-arbitrated damage of a catecholamine attack. The hydro-alcoholic extract of *C. colocynthis* peel also showed cardioprotective potential in experimentally induced myocardial infarction in rabbits, as shown by improvements in histological variations and the estimation of different biochemical and inflammatory markers in injured cardiac tissue. Rabbits pretreated with extract 300 mg/kg for 14 successive days significantly prevented the effect of adrenaline and maintained the biochemical parameters at a normal level (Manzoor et al., 2020).

### 7.10 Neuroprotective Activity

The neuroprotective efficacy of *C. colocynthis* was observed by estimating its effect on endogenous antioxidant molecules in brain samples of a rat with rotenone-induced Parkinson’s disease (Ahmed et al., 2019). The therapeutic impact of *C. colocynthis* and its protective mechanisms confirmed that it showed an excellent neuroprotective impact, lessening oxidative stress and inhibiting apoptotic cell death in both *in-vitro* and *in-vivo* model (Chen et al., 2019). Treatment with hydro-alcoholic *C. colocynthis* pulp extract also showed an anticonvulsant effect in rats. Injection of the *C. colocynthis* extract (25 and 50 mg/kg) exhibited protection against seizure, prolonged the onset of a seizure significantly, and decreased the duration of seizures (Mehrzadi et al., 2016).

All these studies, either *in-vitro* or *in-vivo*, are suggestive of promising effects of *C. colocynthis* and validate its use in traditional medicine as a treatment of gastrointestinal, pulmonary infection and skin infections, constipation, edema, bacterial infections, cancer, diabetes, gastrointestinal disorders, liver problems and as an analgesic.

### 7.11 Toxicity Assessment

The effect of methanolic extract of *C. colocynthis* fruit was evaluated on male albino Wister rats to assess its toxicity. The bone marrow, liver, and kidney functions of the animals were measured using preferred techniques. The acute median deadly dose of the extract was calculated to be 1,311, 45 mg/kg. Plasma AST, urea, ALT, and creatinine titers were affected to a notable extent, indicating that the extract was hepato-nephrotoxic. These findings confirmed that the consumption of the extract of ripe *C. colocynthis* fruit has some undesirable effects on the bone marrow, liver, and kidneys of rats (Soufane et al., 2013).

The membranolytic effect of some *C. colocynthis* components can cause intestinal damage (Javadzadeh et al., 2013). In a study of the subchronic hemotoxicity and cytotoxicity of *C. colocynthis* on albino rats, the oral LD50 for extraction of *C. colocynthis* flowers was found to be 162.4 mg/kg of bodyweight. Pathological adjustments to the lung, liver, kidney, spleen, stomach and intestine of the treated rats were also recorded (Elgerwi et al., 2013). The noxiousness of ingesting an extract with 10% *C. colocynthis* fruits was checked in the rats. The outcomes of *C. colocynthis* treatment were depression, ruffled hair, low body weight, feeding efficiency, and entero-hepato-nephropathy. Diarrhea is a clear sign of *C. colocynthis* poisoning. Lesions were observed on the organs in addition to leukopenia, anemia, modifications in serum enzyme (AST, ALT ALP, and ALT) levels, and concentrations of whole protein, urea, bilirubin, albumin, and one of a kind serum constituent (Al-Yahya et al., 2000). *C. colocynthis* is a strong laxative, with one case report suggesting that ingestion of the former causes inflammation of the colon with bloody diarrhea (Goldfain et al., 1989). High doses of *C. colocynthis* have detrimental effects on liver cells (Dehghan and Panjeh, 2006). High doses of its pulp extract, in particular, were deadly in rabbits, causing dehydration owing to severe diarrhea, heart failure due to cardio-stimulatory action, hepatorenal insults, or hypoglycemia; seed extract caused mild intestinal lesions (Shafaei et al., 2012). Hepatic damage, watery diarrhea, hypoglycemia, and hypotension were observed in a man who received high doses of a *C. colocynthis* fruit decoction to treat constipation (Rezvani et al., 2011). Chickens fed a diet of 10% *Citrullus* developed reversible lesions in their livers, small intestines, and kidneys (Bakhiet and Adam, 1995). Ten sheep fed fresh *C. colocynthis* fruits and leaves developed poisoning symptoms and died between 4 and 25 days of being dosed. Diarrhea, dyspnea, anorexia, and loss of condition are clinical symptoms (Elawad et al., 1984). Oral administration of *C. colocynthis* fruit fruits 0.25 g/kg/day with *Rhazya stricta* leaves resulted in dehydration, loss of condition, profuse diarrhea, ataxia, and recumbency prior to death within 26 days (Adam et al., 2000).

## 8 Applications in Poultry

CCS was fed to 144-day-old straight-run chicks as a potential source of protein in feed, in place of soybean meal. The feeding experiment revealed that including up to 15% of the whole seed in the feed resulted in the normal growth of the chicks. However, the inclusion of 15% unprocessed meals depressed growth and showed a poor feed conversion ratio (FCR) (Sawaya et al., 1986).

CCSP was fed to 360-day-old Ross strain broiler chickens as a 0, 2, 4, and 6% supplement in feed. The result of the study showed that the 6% supplement in feed improved live body weight and dressing percentage while decreasing feed intake and FCR (Ali et al., 2012).

*C. colocynthis* fruit powder was fed to 100 broiler chickens, among which 100 chicks were given this on the sixth day after inoculation with *Eimeria tenella*. The power was supplemented in feed at 0.05, 0.01, 0.15, and 0.00%. The result showed that the 0.15% *C. colocynthis* fruit powder supplement was the most efficient at preventing coccidiosis (ALamery and ALsaeq, 2011). The effects of CCS meal (CCSM) on 270-day-old male Cobb broiler chickens were studied. CCSM was supplemented through feed at 0, 2, and 4%, and results showed that supplementation at 4% improved carcass weight, dressing percentage, and live body weight. As the dietary level of CCSM increased, feed intake decreased, and FCR was impaired (Ali et al., 2012). In another study, *C. colocynthis* was fed to 240-day-old Ross broiler chicks to check the effects of the former on growth performance and intestinal morphology. Here, *C. colocynthis* was supplemented at 0, 0.2, 0.4, and 0.6% of bitter cucumber feed with 0 and 0.01% protein. The results showed that supplementation at 0.6% improved feed intake, body weight gain, breast meat, and carcass yield while reducing FCR. Villus height, crypt depth, and intestinal mucosal muscle also increased (Hashemi et al., 2016).

**FIGURE 5:**
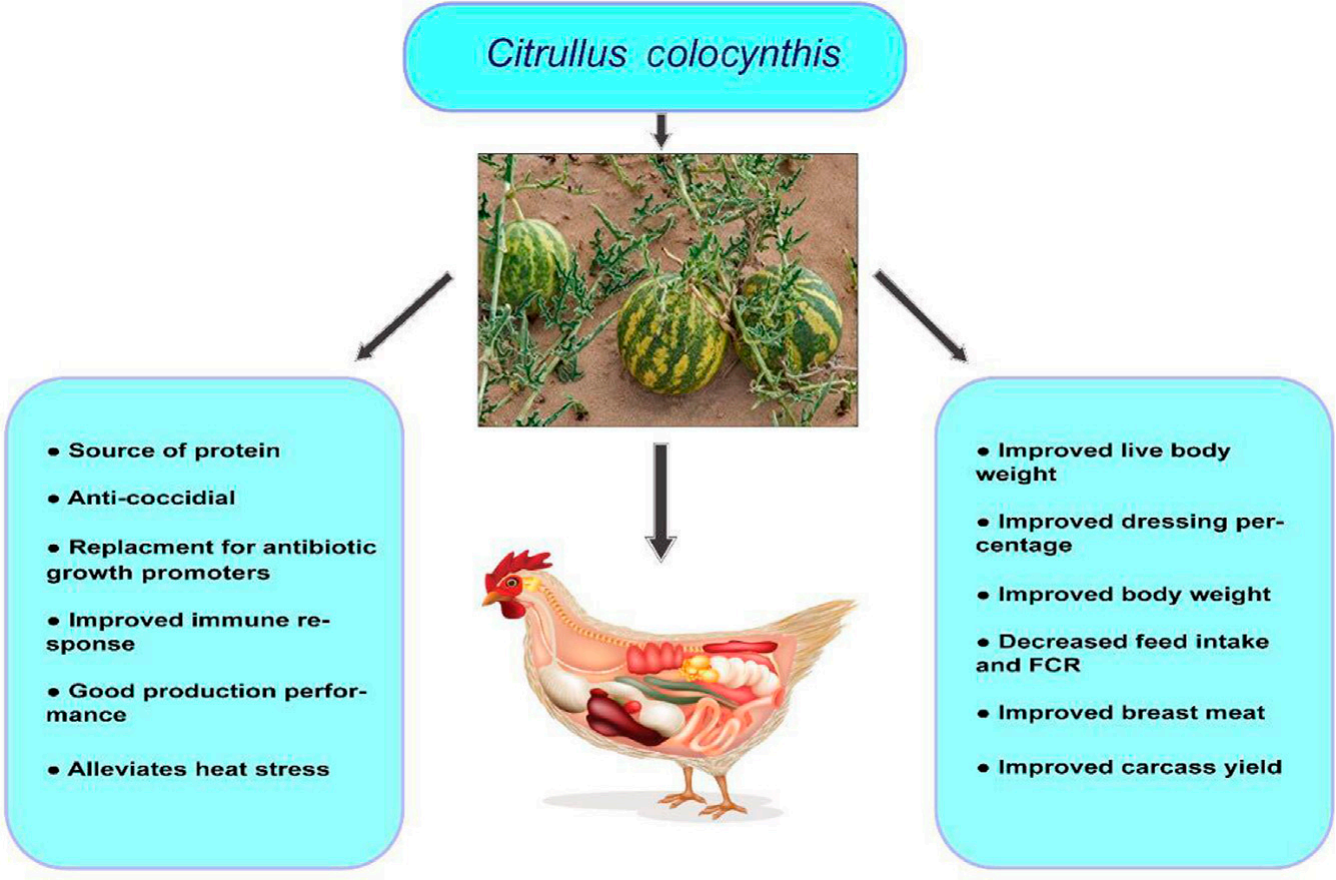
Application of *C. colocynthis* in avian nutrition.

In a different study, *C. colocynthis* fruit pulp was fed to replace antibiotic growth promoters with 400-day-old Ross broiler chicken chicks. Here, *C. colocynthis* fruit pulp was supplemented at 1 g/kg feed and 1.5 g/kg. The result showed that supplementation of *C. colocynthis* fruit pulp at the latter rate could replace antibiotic growth promoters (Kamran et al., 2018). In a separate study, CCS was fed *via* feed to 300 Cobb 21-day-old broiler chickens subjected to chronic heat stress at the rate of 0.1%. The result showed that this supplementation rate improved immune response and production performance in the heat-stressed group but had no effect on a control (thermo-neutral) group (Alzarah et al., 2021). The promising effects of *C. colocynthis* for poultry nutrition are shown in Figure 5.

## 9 Veterinary Uses

At 4 g/day, a polyherbal combination including *C. colocynthis* modified gene expression to promote growth and health from the pre-ruminant to weaning phase. Some gene expression research indicates that polyherbal therapy enhanced lipid, protein, carbohydrate, and immune response metabolism. These findings support using plant chemicals in animal feed (Díaz Galván et al._, 2021). Supplementation with *C. colocynthis* fruit showed potential to minimize methanogenesis and improve rumen fermentation. However, *in vivo* testing on ruminants is required to evaluate the persistence of benefits as well as health issues (Hundal et al., 2020). De-oiled CCS cake was added to dairy cow feed and showed no effect on milk yield (Khatri et al., 1993).

Ten sheep were fed fresh *C. colocynthis* fruits and leaves. The sheep developed poisoning symptoms and died within 4–25 days after being dosed. They showed symptoms of dyspnea, diarrhea, loss of condition, and anorexia (Elawad et al., 1984). *C. colocynthis* fruits oral dosing with *Rhazya stricta* leaves proved deadly within 26 days, resulting in ataxia, profuse diarrhea, loss of condition, dehydration, and recumbency prior to death (Adam et al., 2000). A trial of the ingestion and metabolism of *C. colocynthis* was undertaken in 12 yearlings Najdi sheep to investigate the consumption of crude protein in CCS meal as this was shown to be a good partial substitute for soybean meal in sheep diets (Bhattacharya, 1990). After reviewing the literature, we found that there is currently a lack of research-based data on the use of *C. colocynthis* in veterinary science. More research is needed to determine its importance and note effective inclusion levels in the diet of animals.

## 10 Applications in Humans

The methanolic extract of *C. colocynthis* leaves and its two fractions, ethyl acetate, and chloroform possess notable anti-cancerous effects. Bioassays showed a significant reduction in the multiplication and growth of treated cells compared to untreated cells. Owing to the expression of cyclin-CDK inhibitors, *C. colocynthis* arrests the cell cycle in human breast tumor cells (Perveen et al., 2021). *C. colocynthis* is used for treating colorectal cancer in humans. Cucurbic acid present in *C. colocynthis* extract is believed to stop the multiplication of cancerous cells. The anti-tumor activity of *C. colocynthis* can be attributed to different pathways and effects, such as apoptotic pathways, antioxidant and anti-inflammatory effects, inhibition of Wnt/ß-catenin signaling pathway, and anti-metastatic effects (Abdulridha et al., 2020). CCSP lowers the cholesterol level in non-diabetic patients (Rahbar and Nabipour, 2010). The *C. colocynthis* plant acts as a good anti-diabetic agent in humans with type II diabetes as it reduces glucose and cholesterol levels (Youshan et al., 2015; Chenghe et al., 2014). *C. colocynthis* fruit pulp of mature seed can also be used to treat tuberculosis, and it was found to have active anti-bacterial properties against various strains of normal and drug-resistant *mycobacterium* (Archana et al., 2013). The methanolic extract of *C. colocynthis* fruit is also active against several food-borne bacteria hazardous to human health (Kim et al., 2014). *C. colocynthis* also shows excellent potential as an anti-cancerous agent for treating human breast cancer *via* the modulation of lipid metabolism (Perveen et al., 2020). Consumption of *C. colocynthis* for a long time could lead to anti-fertility issues in both males and females (Chaturvedi et al., 2003; Amal et al., 2016). *C. colocynthis* fruit extract shows anti-tumor activity on drug-resistant cancerous cell lines (Saeed et al., 2019). *C. colocynthis* oil can be used for treating constipation in humans (Qureshi et al., 2010). *C. colocynthis* fruit has shown promising results in treating diabetic patients. A dose of 300 mg/day given to patients showed no adverse effects on their health (Huseini et al., 2009). An extract of *C. colocynthis* leaves can be used to treat skin infections, and a paste of *C. colocynthis* roots can be applied to treat joint problems (Upadhyay et al., 2007). *C. colocynthis* has also been used to treat edema, cancer, constipation, bacterial infections, and diabetes and has been used as an abortifacient (Delazar et al., 2006). *C. colocynthis* is a desert shrub with a long history as a valuable oil source and medicinal plant. During the first 4 weeks of ripening, yield and size of fruit, seed output, and overall oil yield were all at their peak. *C. colocynthis* has the potential to be grown as a source of edible oil. The oil has a laxative effect and contains between 80 and 85 percent unsaturated oleic and linoleic fatty acids, making it a high-quality oil for human consumption (Schafferman et al., 1998).

**FIGURE 6:**
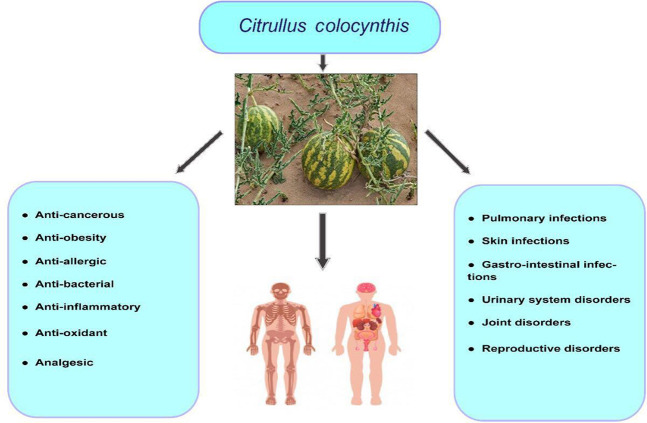
Health benefits of *C. colocynthis* in humans.

The health benefits of *C. colocynthis* are shown in Figure 6.

## 11 Limitation and Future Research

The chemical cucurbitacin, found in colocynth, irritates mucous membranes, especially those in the stomach and intestines. Colocynth is extremely dangerous to use. The Food and Drug Administration (FDA) prohibited it in 1991. Even tiny doses of colocynth can induce severe stomach and gut lining irritation, bloody diarrhea, kidney damage, bloody urine, and inability to pee. Convulsions, paralysis, and death are some of the other adverse effects. Colocynth dosage is determined by a number of factors, including the user’s age, health, and other circumstances. There is insufficient scientific evidence to define a suitable dosing range for colocynth. Keep in mind that natural products aren’t always safe and that doses are crucial. Before using, be sure to read the product label and consult your pharmacist, physician, or another healthcare expert. According to reports, ingestion of merely 1-1/2 tablespoons of the powder has resulted in death. Colocynth is unsuitable for use during pregnancy and breastfeeding. Ingestion of less than 2 gm of the powder has been reported to result in death. In clinical studies, as little as 300 mg of colocynth powder was found to cause moderate diarrhea.

Oral administration of *C. colocynthis* fruits 0.25 g/kg/day with Rhazya stricta leaves proved deadly within 26 days, resulting in dehydration, loss of condition, profuse diarrhea, ataxia, and recumbency before death (Adam et al., 2000). The treatment regimen having 10% of *C. colocynthis* fruits was once checked for rats. The outcomes characterized by *C. colocynthis* treatment were depression, ruffled hair, low physique weight, low feed efficiency, and entero-hepato-nephropathy (Al-Yahya et al., 2000). Chickens fed a 10% Citrullus diet developed reversible lesions in the liver, small intestine, and kidney (Bakhiet and Adam, 1995). 60 ml of decoction of the plant fruit taken by a 48-year old man to treat constipation resulted in watery diarrhea, hypotension and hypoglycemia, and hepatic injury (Rezvani et al., 2011). *C. colocynthis* being stimulant laxatives can cause the body’s potassium levels to drop. Low potassium levels might exacerbate digoxin side effects. Lanoxin: Colocynth can induce diarrhea in some patients who are taking Warfarin. Diarrhea can make Warfarin less effective and raise the risk of bleeding. Taking colocynth with water pills may cause the body’s potassium levels to drop too low.

The biological activity of the extracts and isolated compounds have been discovered, particularly in antidiabetic, anticancer, anti-inflammatory, antioxidant, insecticidal, and antibacterial areas. Interestingly, the plant has been demonstrated to have a high nutritional value since it is a strong source of protein, has edible seed oil, and contains certain vital minerals such as calcium, potassium, and magnesium, all of which are known to have medical benefits. Despite the fact that growing interest has driven greater research on *C. colocynthis*’ phytochemistry and pharmacology, there are still many areas where existing understanding might be improved. Furthermore, there is a scarcity of information concerning its mode of action and dosing rate. In recent pharmacological investigations, various traditional applications of the *C. colocynthis* fruit have been verified; however, some of these studies were only examined *in vitro*. As a result, *in vivo* experiments should be used to evaluate further the efficacy and safety of *C. colocynthis* fruit extracts and isolated chemicals. *In previous studies, C. colocynthis* has been shown to have many roles in people, cattle, and fowl. The aforementioned system’s literature has been evaluated from a variety of sources. Because there is limited research on the use of *C. colocynthis* in poultry, veterinary medicine, and human medicine, it is the forward reassessment to advocate *C. colocynthis* plant and extract for use in poultry and human medicine manufacturing.

## 12 Conclusion and Perspectives

In the present assessment, the nutrient composition and medicinal qualities of *C. colocynthis* have been evaluated based on various previous studies. This review strongly indicates that *C. colocynthis* is a fruit crop that could benefit the treatment of a range of diseases. Although *C. colocynthis* has high dietary value, it is not widely known. More investigations are required to spotlight the utility of such fruit crops as a dietary supplement that can enhance fitness. This review demonstrates that *C. colocynthis* is a medicinal plant with a wide variety of pharmacological properties that might make it useful and effective in numerous medical applications. To date, no review article has published comprehensive literature about its uses in poultry, veterinary and human areas. This review gives a thorough insight into its phytochemistry along with the structure-activity relationship of some bioactive compounds, pharmacology, beneficial effects, limitations, and drug interaction. The study compiles the most recent data present on *C. colocynthis*. So, the objective of this review is to provide comprehensive data about the benefits and limitations of *C. colocynthis*, as the data about inclusion levels and its use and possible side effect are still not precise and need to be validated by pharmacological investigations against various disorders *in vivo*. *C. colocynthis* has many vital health-promoting effects like neurological, physiological as well as biological functions, but still, their mechanism of action behind these properties in different species is not known and needs to be exploited. The future avenues for the veterinary and pharmaceutical researchers would be to identify more of these demanding areas and document reliable markers (bio and molecular) which are responsible for a vast array of *C. colocynthis’s* benefits.
